# Research on the correction method for radiotherapy verification plans based on displaced electronic portal imaging device

**DOI:** 10.1002/acm2.14401

**Published:** 2024-05-22

**Authors:** Jian Guo, Leyuan Zhou, Haibin Zeng

**Affiliations:** ^1^ Department of Radiation Oncology The First Affiliated Hospital of Soochow University Suzhou China; ^2^ Department of Radiation Oncology The Fourth Affiliated Hospital of Soochow University Suzhou China

**Keywords:** electronic portal imaging device (EPID), plan verification, portal dosimetry (PD)

## Abstract

**Background:**

It has been observed that under the single isocenter conditions, the potential shifts of the electronic portal imaging devices (EPID) may be introduced when executing portal dosimetry (PD) plans for bilateral breast cancer, pleural mesothelioma, and lymphoma. These shifts are relative to the calibration positions of EPID and result in significant discrepancies in the plan verification results.

**Purpose:**

To explore methods including correction model and specific correction matrices to revise the data obtained from displaced EPID.

**Methods:**

Two methods, the correction model and the specific correction matrices, were applied to correct the data. Five experiments were designed and conducted to build correction model and to validate the effectiveness of these two methods. Gamma passing rates were calculated and data profiles along X‐axis and Y‐axis were captured.

**Results:**

The gamma passing rates for the EPID‐displaced IMRT validation plans after applying correction model, along with the application of specific correction matrices to VMAT and IMRT validation plans, exhibit results that are comparable to the cases with non‐displaced EPID. Except for the VMAT plans applied correction model which showed larger discrepancies (0.041 ± 0.028, 0.049 ± 0.030), the other three exhibit minimal differences in discrepancy values. In all profiles, the corrected data from displaced EPID exhibit a high level of agreement with data obtained from non‐displaced EPID. Good consistency is observed in actual application of the correction model and the specific correction matrices between gamma passing rates of data corrected and those of non‐displaced data.

**Conclusions:**

The proposed methods involving correction model and specific correction matrices can correct the data collected from the displaced EPID, and the gamma passing rates of the corrected data show results that are comparable to some extent with those of non‐displaced data. Particularly, the results corrected by specific correction matrices closely resemble the data from non‐displaced EPID.

## 1 INTRODUCTION

The plan quality control is a crucial step to ensure the safety of radiotherapy before a patient undergoing treatment, which requires a series of methods and devices to validate the feasibility of the treatment plan and the reliability of the output dose. In comparison to films and other third‐party verification devices, the amorphous silicon electronic portal imaging devices (EPID) installed on the linear accelerator, outputs digital signals directly, possesses advantages such as high resolution and efficient process execution. Therefore, it has been widely applied in accelerator quality control, position verification, and dose verification.^1^, ^2^

In daily work, it has been observed that under the single isocenter condition, the potential shifts of the EPID may be introduced when executing portal dosimetry (PD) plans for bilateral breast cancer, pleural mesothelioma, and lymphoma. These shifts are relative to the calibration positions of EPID and result in significant discrepancies in the plan verification results. Additionally, the shifts of the EPID during PD verification may occur in certain cases where the target regions approach or extend beyond the boundaries of EPID, like plans for cervical and esophageal cancer with elongated target regions and large collimator angles, or those involving special postural and treatment considerations. This anomaly leads to abnormal plan verification and extremely low pass rates, which significantly impacts the validation of patients' radiotherapy plans.

**FIGURE 1:**
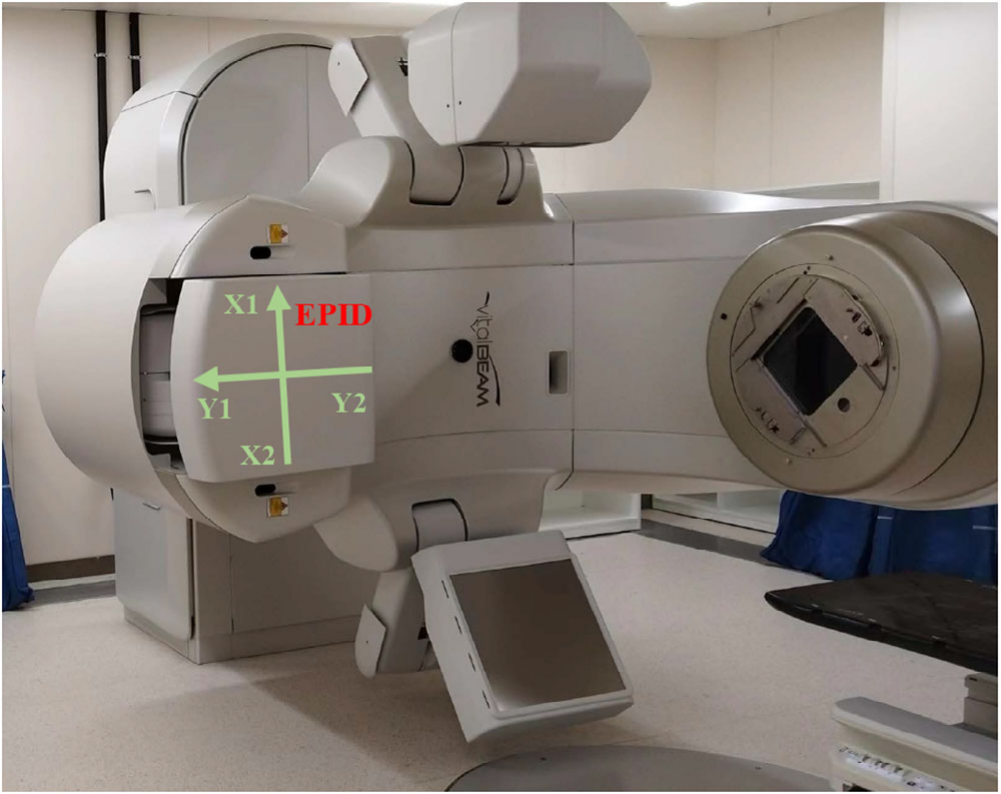
Instrumentation, including the Varian VitalBeam accelerator and the onboard EPID. The green arrows indicate the movement directions of the EPID, and the directions of the arrows point in the positive directions.

In the preliminary testing phase, open‐field plans are designed under field sizes of 15 cm × 15 cm and 25 cm × 25 cm, with the EPID offset 2 cm towards X1, X2, Y1, and Y2 (directions are indicated in Figure 1), respectively. Corresponding PD verification plans have been generated and conducted. The results indicate that there is a substantial error in EPID data exported from the system. The data consistently shows that after moving the EPID panel 2 cm along the Y‐axis direction towards Y1, the Y‐axis profile tilts towards Y1 (profile defined as Y‐Y1), with lower Y1 and higher Y2. Conversely, tilting in the opposite direction occurs after moved 2 cm towards Y2 (profile defined as Y‐Y2). In both cases, X‐axis profiles remains smooth. Additionally, profile Y‐Y1 and Y‐Y2 are symmetrical along the vertical axis where the curves intersect. The same result is also observed when moving the EPID panel along the X‐axis. Integrated into the Varian onboard system, the aSi‐1200 MV EPID panel used in this study is designed with a backscatter shielding layer, eliminating the need for backscatter correction.^3^ Consequently, the radiation field center data obtained by the detector should not vary significantly after moving 2 cm.

Presently, to the best of our knowledge, no research has proposed a suitable solution to address this issue. Considering that the regularity observed in the preliminary results, data calibration is worth further investigation. Therefore, this study aims to explore a correction model and specific correction matrix methods to correct the data obtained from the displaced EPID.

## 2 METHODS

### 2.1 Patient information and equipment

Twenty patient plans from September 2021 to November 2022 were selected, which were carried out in our radiotherapy department and satisfied the criterion that the coronal target area was longer than 17 cm. These plans included eleven volumetric modulated arc therapy (VMAT) plans with 2 arcs, and nine intensity‐modulated radiation therapy (IMRT) plans with 4 fields. All plans used 6 MV x‐rays with a dose rate of 600 MU/min. The Anisotropic analytical algorithm and portal dose image prediction (PDIP) algorithm were employed for dose calculation and prediction, respectively. All PD verification plans in this paper were generated under a source imager distance of 100 cm, the same as the calibration position of the EPID panel.

The linear accelerator involved in this study is the Varian VitalBeam, equipped with the Eclipse planning (version 15.6) and treatment (version 2.7) systems. The multi‐leaf collimator (MLC) is Millennium 120, with a size of 40 cm × 40 cm. The onboard aSi‐1200 MV EPID panel has an effective measurement area of 40 cm × 40 cm and a pixel pitch of 0.0336 cm.

### 2.2 Method description

A correction matrix is applied to correct the data captured by the displaced EPID. To acquire the correction matrix, a correction model is constructed in advance by collect the EPID panel data of different field sizes and different displacements. Specifically, the data captured from the non‐displaced and displaced EPID were set as the target and input of the correction model, respectively. The resulting data, target divided by input, yielded to different correction matrices for various field sizes and displacement distances. To simplify the correction process, all collected EPID data were designed as square fields.

Currently, the generated correction model is only applicable to square fields and lacks correction applicability to rectangular fields. Therefore, besides constructing a correction model to obtain a correction matrix, this study has also explored whether it is possible to customize a specific correction matrix based on field conditions for the revision of data from displaced EPID. To achieve this, EPID data with and without displacement for open fields with the same sizes are required. The ratio of these two data sets is the customized correction matrix, which is suitable for the corresponding field size and displacement distance, and is applied to correct data for a specific patient's PD verification plan.

The following formula describes the relationship among the data obtained from the displaced ($A_{f,d}$) and non‐displaced ($B_{f,d}$) EPID as well as the correction matrix $T_{f,d}$.

(1) $$A_{f,d}\cdot \;T_{f,d}=B_{f,d}\;$$

Here, the subscripts *f* and *d* represent the field sizes and displacement distances, respectively and the $T_{f,d}$ is generated from the correction model or the specific correction matrices.

### 2.3 Experimental setup

Table 1 describes the experimental setups. In the correction model construction experiment (Experiment No.1), open‐field plans with field sizes of 5 cm × 5 cm, 10 cm × 10 cm, 15 cm × 15 cm, 20 cm × 20 cm, 25 cm × 25 cm, and 30 cm × 30 cm were designed. Based on these open‐field plans, PD verification plans with EPID displaced 0.0 cm (i.e., without displacement), 0.5, 1, 1.5, 2, 2.5, 3, 3.5, 4, and 4.5 cm were created and conducted. A correction model based on the above data was generated.

**TABLE 1:** Experimental setups.

No.	Field type	Field size (cm)	Note for field size	Displacement (cm)	Purpose
1	Open field	5 × 5	–	0, 0.5, 1.0, 2.0, 2.5, 3.0, 3.5, 4.0, 4.5	Generate correction model
		10 × 10			
		15 × 15			
		20 × 20			
		25 × 25			
		30 × 30			
2	Open field	5 × 5	–	2.2	Validate the effectiveness of correction model of different displacement and other field sizes
		10 × 10			
		15 × 15			
		20 × 20			
		25 × 25			
		30 × 30			
		12.5 × 12.5		2.2, 3.7	
		27.5 × 27.5			
3	Fields of treatment plans with MLC, a VMAT plan, a IMRT plan	5 × 5	Both for IMRT and VMAT	0, 2.2, 3.7	Validate the correction model's effectiveness for actual field with MLC
		10 × 10			
		12.5 × 12.5			
		15 × 15			
		20 × 20	VMAT only		
		25 × 25			
		27.5 × 27.5			
		30 × 30			
4	Fields of treatment plans with MLC, a VMAT plan, a IMRT plan	15 × 5	Both for IMRT and VMAT	0, 3.7	Validate the specific correction matrix's effectiveness for actual field with MLC
		15 × 10			
		15 × 15			
		15 × 20			
		15 × 25			
		15 × 30			
5	Application in actual plans, 10 VMAT plans, 8 IMRT plans	Fit to target with a 0.6 cm margin	It is rounded to nearest integer when applied correction model	0, one of the three displacement (3.7, 4.0, 4.2) for certain plan	Validate the effectiveness of correction model and specific correction matrix for actual plans

The correction model validation experiment (Experiment No.2) contained two stages. In stage one, where the EPID is offset 2.2 cm, PD verification plans for open‐field plans with sizes of 5 cm × 5 cm, 10 cm × 10 cm, 15 cm × 15 cm, 20 cm × 20 cm, 25 cm × 25 cm, and 30 cm × 30 cm were designed and conducted, considering as the actual correction matrix. These data were collected to validate the effectiveness of the correction model for existing field sizes at different displacements.

In stage two, open‐field plans with sizes of 12.5 cm × 12.5 cm and 27.5 cm × 27.5 cm were designed. Then, PD verification plans for EPID panel positions displaced 0.0 cm (without movement), 2.2 cm and 3.7 cm were generated. These data were collected to validate the effectiveness of correction model for other square field sizes.

Experiment No.3 was designed to validate the correction model's effectiveness for fields of treatment plans with MLC, containing one patient using VMAT and one patient using IMRT. For VMAT plans, eight plans with different field sizes were generated under fixed jaws. Field sizes were adjusted to 5 cm × 5 cm, 10 cm × 10 cm, 12.5 cm × 12.5 cm, 15 cm × 15 cm, 20 cm × 20 cm, 25 cm × 25 cm, 27.5 cm × 27.5 cm, and 30 cm × 30 cm. Then, corresponding PD verification plans for EPID panel positions displaced 0.0, 2.2, and 3.7 cm were generated. Due to limitations of MLC, the IMRT plan generated four plans with different field size under fixed jaws. Field sizes were adjusted to 5 cm × 5 cm, 10 cm × 10 cm, 12.5 cm × 12.5 cm, and 15 cm × 15 cm. Similarly, corresponding PD verification plans for EPID panel positions displaced 0.0, 2.2, and 3.7 cm were generated.

Experiment No.4 was designed to validate the specific correction matrix's effectiveness for fields of treatment plans with MLC. The same VMAT and IMRT plans selected in Experiment No.3 were used, generating six plans with different field sizes for each treatment plan. Field sizes were adjusted to 15 cm × 5 cm, 15 cm × 10 cm, 15 cm × 15 cm, 15 cm × 20 cm, 15 cm × 25 cm, and 15 cm × 30 cm. Corresponding PD verification plans for EPID panel positions displaced 0.0 and 3.7 cm were generated. Otherwise, to obtain specific correction matrices, corresponding open‐field plans and PD verification plans with sizes of 15 cm × 5 cm, 15 cm × 10 cm, 15 cm × 15 cm, 15 cm × 20 cm, 15 cm × 25 cm, and 15 cm × 30 cm were designed and generated with a dose of 100 MU.

**FIGURE 2:**
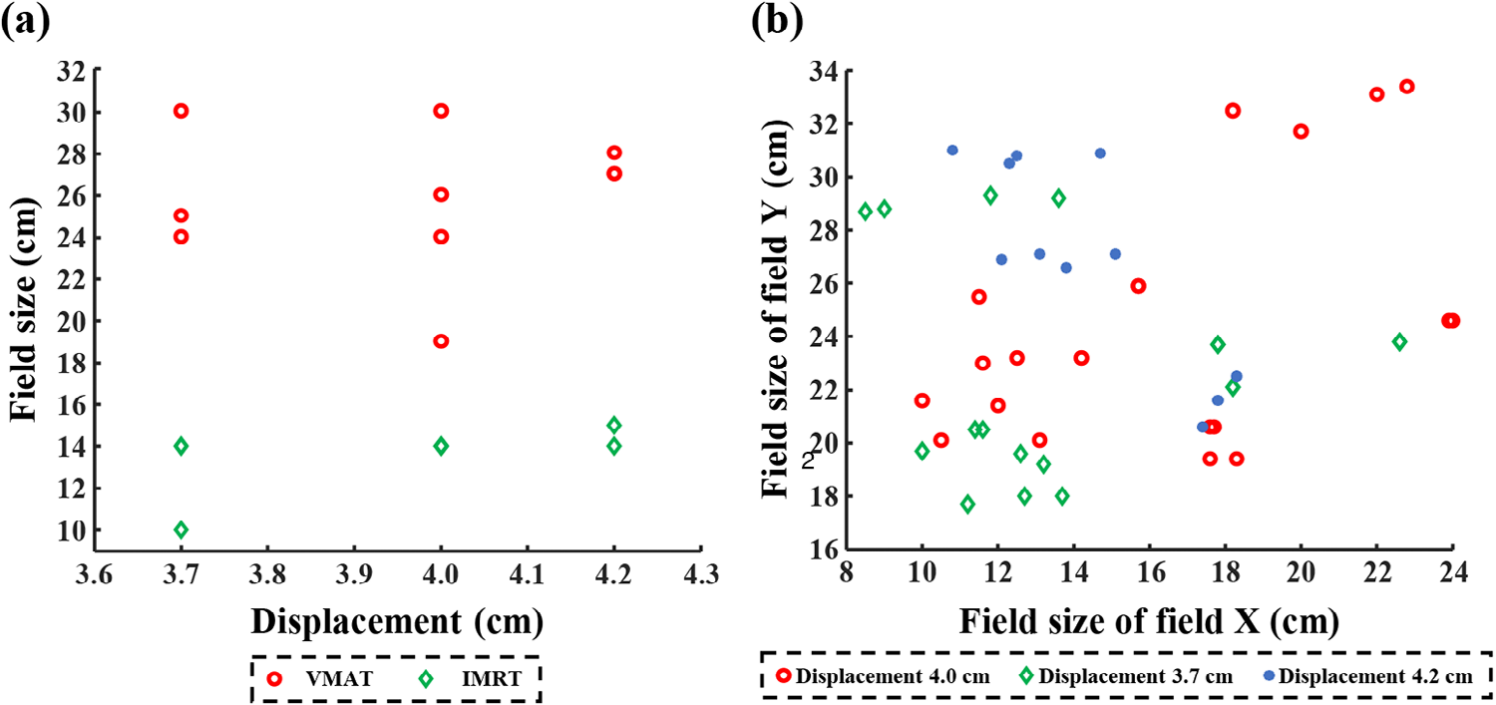
Field sizes of generated plans in Experiment No.5 for the validation of the effectiveness of (a): the correction model, and (b) the specific correction matrices.

Experiment No.5 was designed to validate the effectiveness of the correction model and specific correction matrices for actual treatment plans. Eighteen past patients were selected, including ten using VMAT and eight using IMRT. Three plans were created with fixed jaw for each patient, the first plan was designed to validate the correction model, the second plan was designed to validate the specific correction matrices and the last plan was an open‐field plan, which is corresponding to the second plan and aiming to get the correction matrix. Field size was fitted to target with a 0.6 cm margin. Separately, when generating plans for validating the correction model, field sizes are kept square with the side length rounded to the nearest integers after fitting to target areas. Field sizes of plans used in this experiment were shown in Figure 2. As for the displacement of the EPID, data obtained at the offset distance of 0 cm and one of three larger distances (3.7, 4.0, and 4.2 cm), which was more common in practically, were collected for all patients. Afterward, PD verification plans with different displacements were created.

It should be noted that the doses set in Experiments No.1 and No.2 were 100 MU. To simplify the correction process, the collimator angle was zeroed for VMAT plans in Experiments No.3, No.4, and No.5. Besides, the gantry and collimator angles were zeroed for IMRT plans.

### 2.4 Data collection and processing

After performing PD calibration, PD verification plans for all five experiments were executed on the accelerator. EPID data for each field was collected. Predicted dose images calculated by the PDIP algorithm in the system were exported as reference predicted doses.

All data were processed through Matlab R2020a (The MathWorks, Natick, MA, USA). Due to the discrepancy in dimensions and resolutions between the exported reference predicted dose data (40.2 cm × 40.2 cm, 1024 × 1024) and the EPID panel measurement data (40 cm × 40 cm, 1190 × 1190), a bicubic interpolation method was applied to increase the resolution of the predicted dose data by a factor of 1.168. Then, the central part of 1190 × 1190 data was selected as the reference. Additionally, considering the maximum movement of the EPID panel was 4.5 cm, the calculation range was limited to data within the central 31 cm × 31 cm of the field size, with a resolution of 922 × 922.

**FIGURE 3:**
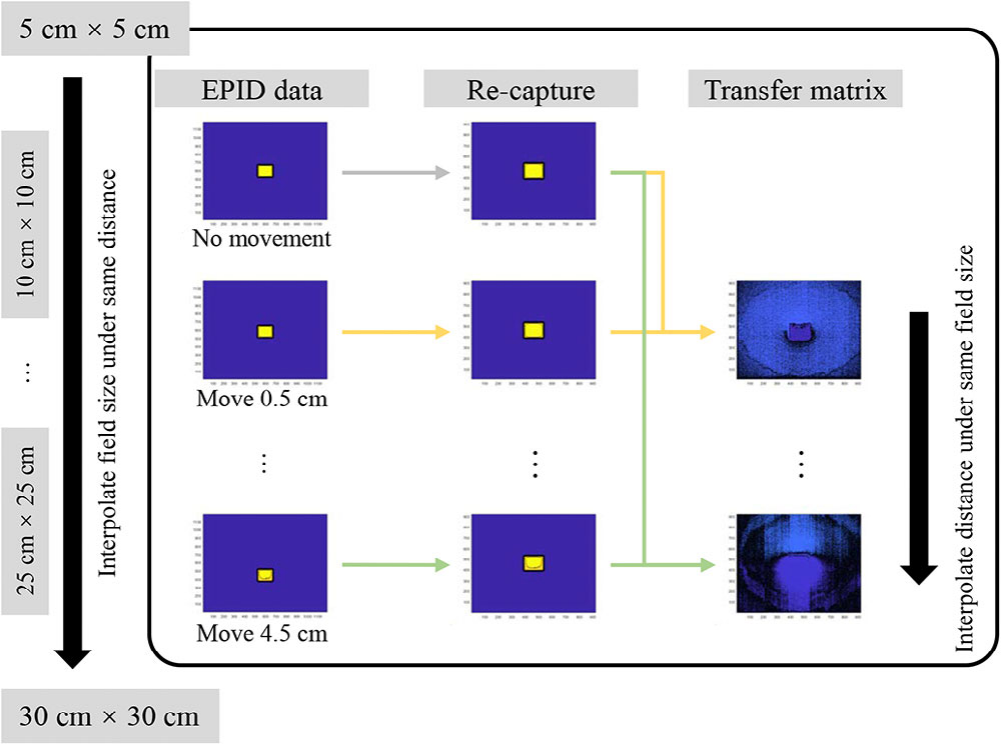
The construction workflow of the correction model.

As shown in Figure 3, for Experiment No. 1, the correction matrices for varying displacement distances were obtained by bicubically interpolating the corresponding matrices for discrete offsets of EPID, which were calculated from the data with the same field size. For the interpolation workflow of data with the same displacement, the data were enlarged for sizes of 5 cm × 5 cm, 10 cm × 10 cm, 15 cm × 15 cm, 20 cm × 20 cm, 25 cm × 25 cm, and 30 cm × 30 cm by factors of 6, 5, 4, 3, 2, and 1, respectively. All data were taken from the central area with 922 × 922 pixels and a bicubic interpolation method was applied to interpolate data of different field sizes. The interpolated data were reduced by corresponding factors, that for 12.5 cm × 12.5 cm and 27.5 cm × 27.5 cm were 2.4 and 1.09, respectively. The data were then interpolated and extended to a size of 922 × 922 through upper and lower layer data. Through these two interpolations, correction matrix data for the correction model were constructed, covering field sizes from 5 cm × 5 cm to 30 cm × 30 cm and displacement distances from 0 to 4.5 cm. Correction model was performed by searching the table based on field size and displacement distance.

For Experiment No.2, data obtained without EPID movement were divided by those collected from displaced EPID (the offset equals 2.2 cm) to obtain the actual correction matrix for the corresponding square field size. Finally, the gamma passing rates were calculated between the actual correction matrix and the interpolated correction matrix obtained from the correction model.

As for Experiments No.3, No.4 and No.5, the gamma passing rates between the data corrected by the correction model or specific correction matrix and the reference predicted dose were calculated for VMAT and IMRT plans, considering both displaced and non‐displaced scenarios. Both scenarios used a 2%/3 mm standard with a dose threshold of 5%. Additionally, for Experiments No. 3 and No. 4, the average and standard deviation (STD) values of gamma passing rates of data corrected by the above two different correction methods, were calculated. Further, the average and STD values of the gamma passing rate differences between the corrected displaced and original non‐displaced data, as well as the sum of squares for error (SSE) of the central data profiles on the X‐ and Y‐axis were also obtained for these two experiments.

### 2.5 Statistical analysis

Utilizing paired‐sample *t*‐tests, statistical analysis is conducted between the gamma passing rates of the data corrected and the gamma passing rates of the non‐displaced data. Bland‐Altman plot is applied to results of Experiment No.5 for the consistency test. SPSS 26.0 (SPSS Inc., Chicago, IL, USA) is utilized for all statistical analyses, and *p* < 0.05 is considered as significant difference.

## 3 RESULTS

Table 2 shows the results of the gamma passing rates between correction matrices obtained from the correction model and actual correction matrices, under the condition that the EPID displaced 2.2 cm for open‐field plans with sizes of 5 cm × 5 cm, 10 cm × 10 cm, 12.5 cm × 12.5 cm, 15 cm × 15 cm, 20 cm × 20 cm, 25 cm × 25 cm, 27.5 cm × 27.5 cm, and 30 cm × 30 cm. The results indicate that the correction model exhibits extremely high gamma passing rates compared to the actual correction matrices (gamma passing rate > 0.96). Noteworthy, gamma passing rate in 12.5 cm × 12.5 cm is lower compared to others.

**TABLE 2:** Results of the gamma passing rates between correction matrices obtained from the correction model and actual correction matrices.

Square field size (cm)	5	10	12.5	15	20	25	27.5	30
Gamma passing rate	0.997	0.999	0.968	1.000	1.000	0.999	1.000	1.000

**FIGURE 4:**
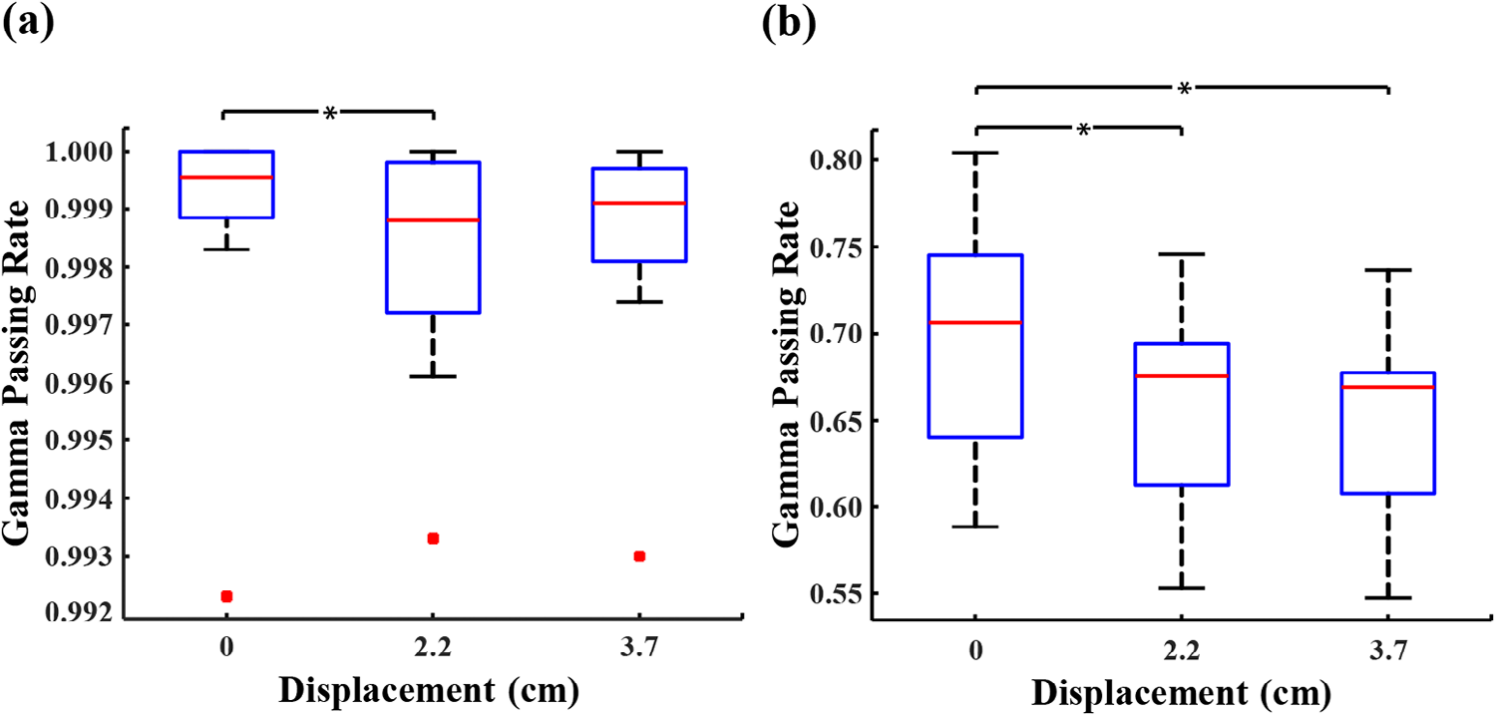
Gamma pass rates between predicted dose and measured dose for PD verification plans after applying the correction model. (a) gamma passing rates for IMRT verification plans, (b) gamma passing rates for VMAT verification plans. * represents *p* < 0.05.

Figure 4 shows the gamma passing rates between the predicted dose and the measured dose for PD verification plans in Experiment No.3. Clearly, for the gamma passing rates of VMAT verification plans in Figure 4b, a significant difference is observed between corrected data from the displaced EPID and original data from the non‐displaced EPID. For different offset distances of 2.7 and 3.7 cm, the results of statistical analyses are *t* = 5.721, *p*< 0.05 and *t* = 6.594, *p* < 0.05, respectively. In Figure 4a, similar analyses are presented for IMRT verification plans, where significant and insignificant differences are observed with the EPID displaced 2.2 cm (*t* = 2.473, *p* = 0.026) and 3.7 cm (*t* = 2.111, *p* = 0.052), respectively.

**FIGURE 5:**
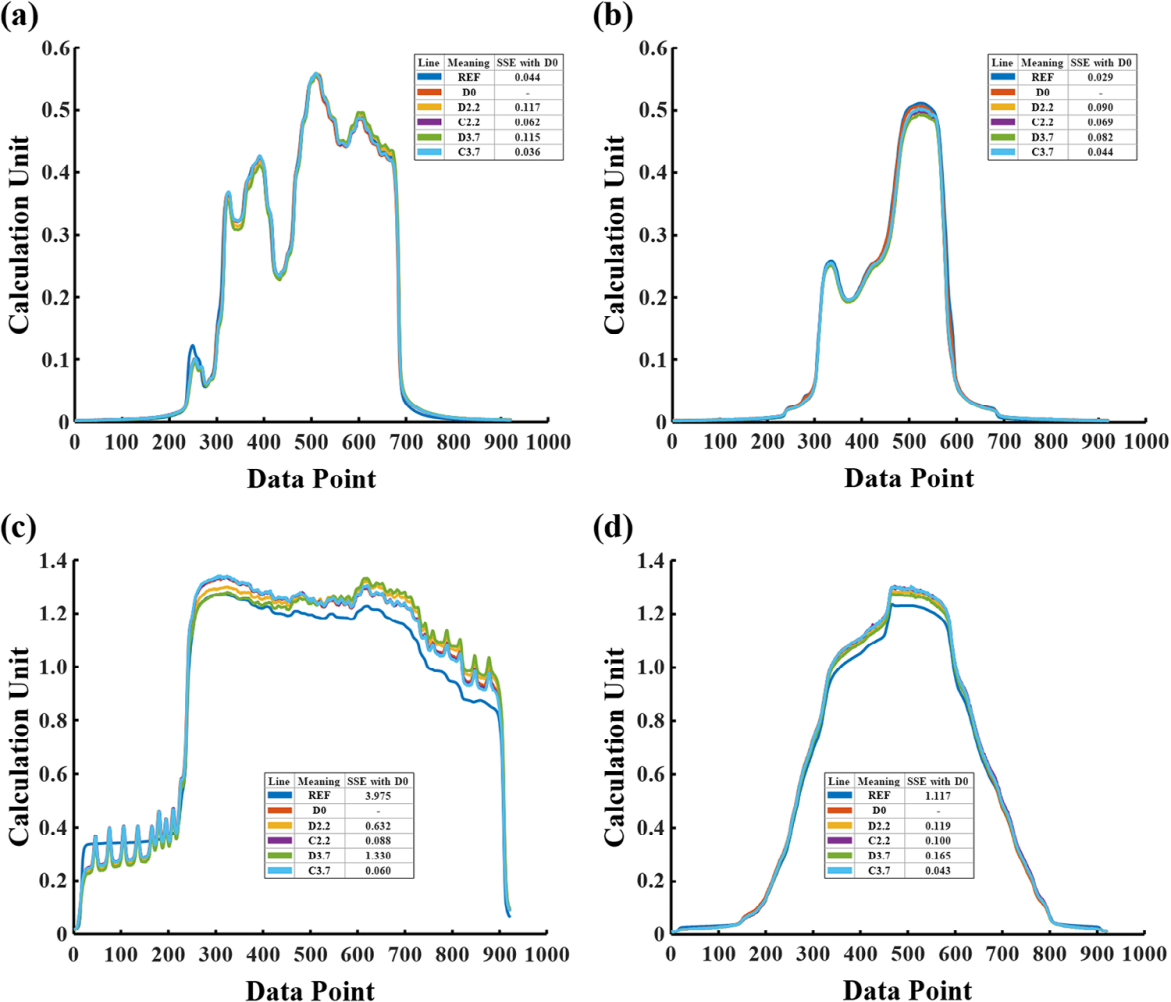
The central data profiles on the X‐ and Y‐axis and SSE values for IMRT and VMAT validation plans, when the correction model is applied to the actual verification plan. (a) and (b) are the central data profiles on the X‐ and Y‐axis captured from IMRT validation plans under conditions of a field size of 15 cm × 15 cm and the EPID displaced 2.2 cm and 3.7 cm. (c) and (d) are similar to (a) and (b) respectively, but for VMAT validation plans where the field size of 30 cm × 30 cm is used.

Figure 5 depicts the central data profiles on the X‐ and Y‐axis and SSE values for IMRT and VMAT validation plans after applying the correction model to the actual verification plan. Results from Figure 5a and Figure 5b show major differences (SSE > 0.08) in EPID data acquired under a displacement of 2.2 cm (D2.2) and EPID data acquired under a displacement of 3.7 cm (D3.7) compared to EPID data acquired without displacement (D0), both in the X‐axis and Y‐axis profiles for IMRT validation plans. In contrast, the data corrected under a displacement of 2.2 cm (C2.2) and 3.7 cm (C3.7) exhibit a high level of agreement with D0 (SSE < 0.07 and shows lower values). Similar results can be observed in results of VMAT validation plans. However, in IMRT validation plans, the predicted reference dose calculated by the PDIP algorithm (REF) is closer to the D0 (SSE = 0.044, 0.029) where a notable difference (SSE = 3.975, 1.117) is observed in VMAT validation plans.

**FIGURE 6:**
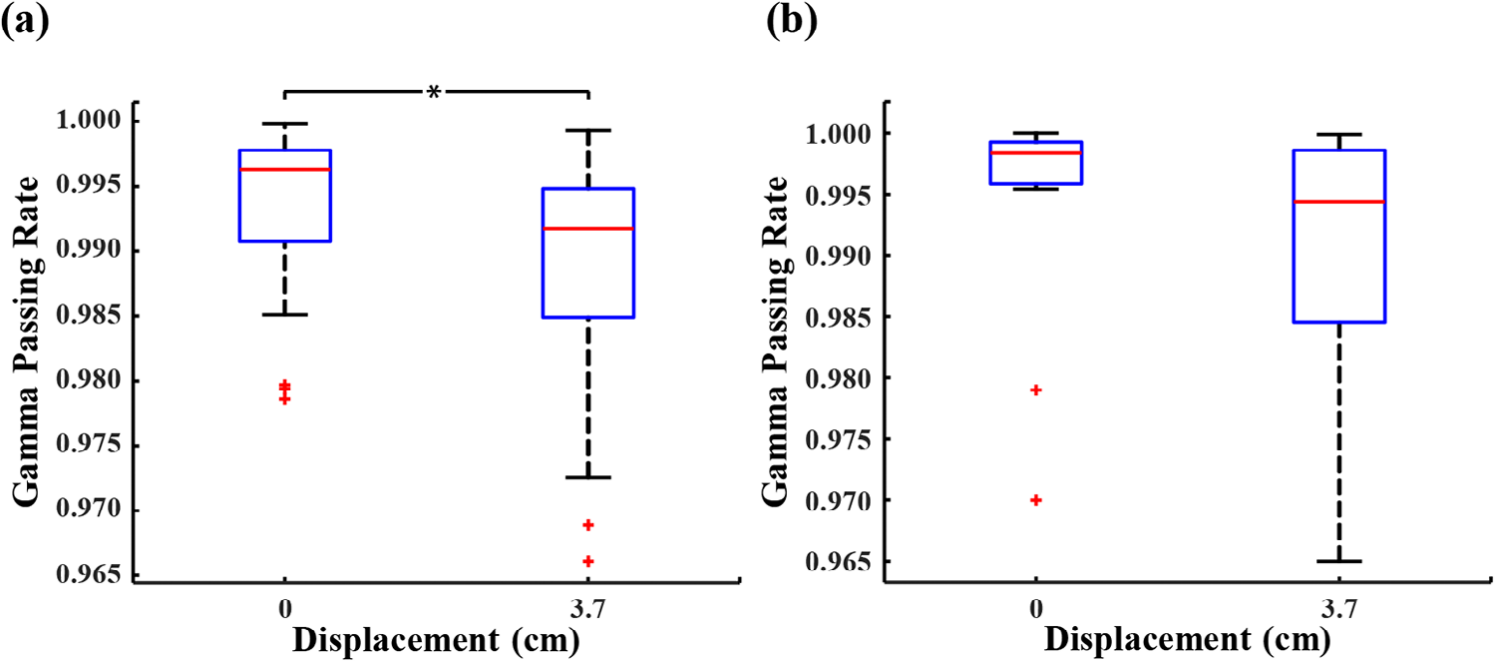
Gamma pass rates between predicted dose and measured dose for PD verification plans after applying the specific correction matrix. (a) gamma passing rates for IMRT verification plans, (b) gamma passing rates for VMAT verification plans. * represents *p* < 0.05.

Figure 6 illustrates the gamma passing rates between the predicted dose and the measured dose for PD verification plans in Experiment No.4. As illustrated in Figure 6b, for VMAT verification plans, no significant difference (*t* = 1.45, *p* = 0.175) is observed between the gamma passing rates of the corrected data, which are collected by the EPID displaced a distance of 3.7 cm, and that of the non‐displaced data. However, there is a significant difference (*t* = 7.907, *p* < 0.05) between the gamma passing rates when the IMRT verification plans are used, as shown in Figure 6a.

**FIGURE 7:**
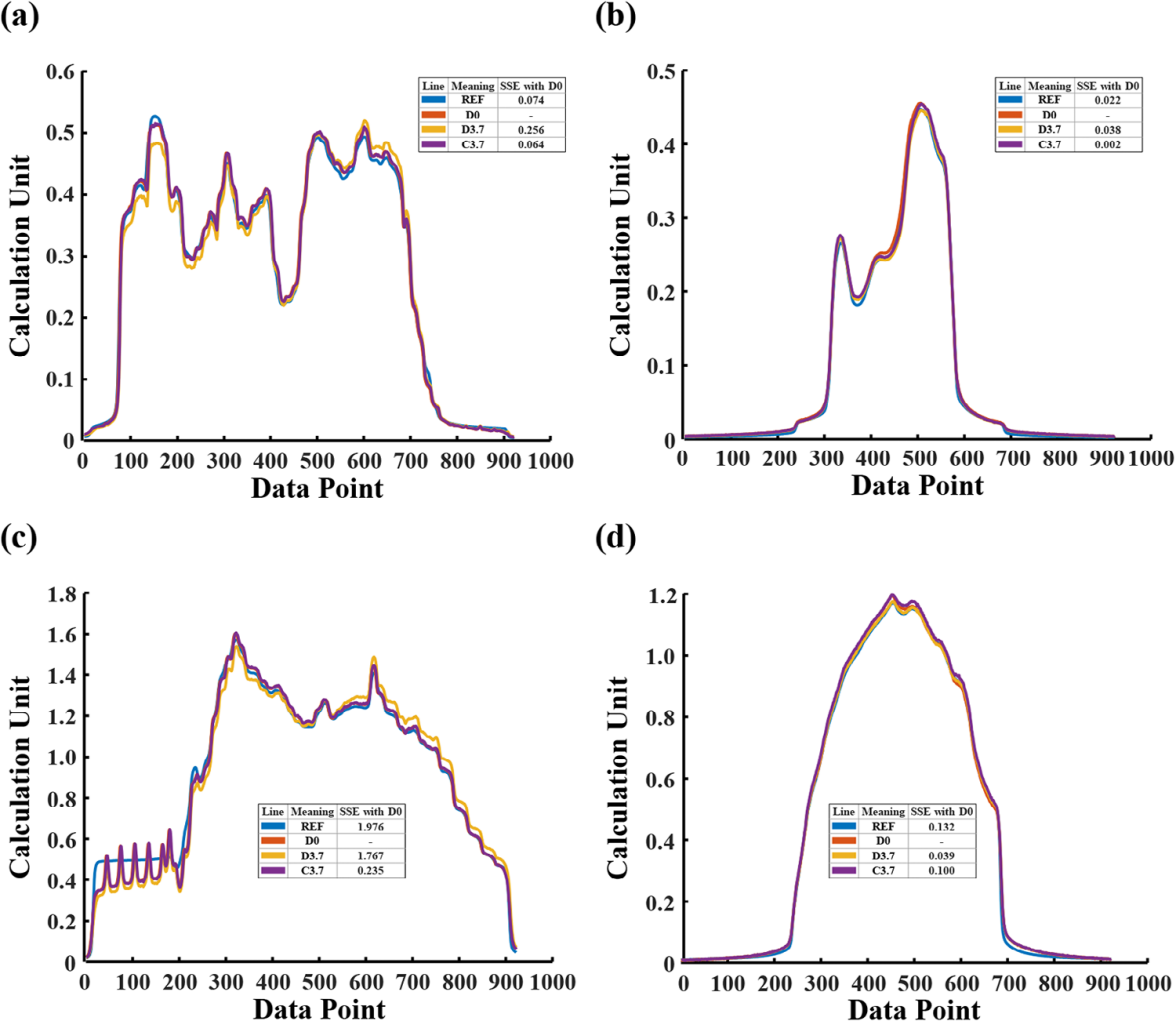
The central data profiles on the X‐ and Y‐axis and SSE values for IMRT and VMAT validation plans after applying a specific correction matrix to the actual verification plan. (a) and (b) are the central data profiles on the X‐ and Y‐axis captured from IMRT validation plans under conditions of a field size of 15 cm × 30 cm and the EPID displaced 3.7 cm. (c) and (d) are similar to (a) and (b) respectively, but for VMAT validation plans.

Figure 7 is similar to Figure 5 but the corrected data are revised by applying the specific correction matrix and a field size of 15 cm × 30 cm is used. Results from Figure 7a and Figure 7b reveal that there are major differences between the data D3.7 and D0 for IMRT validation plans (SSE = 0.256, 0.038), while the corrected data C3.7 demonstrates a high level of agreement with D0 (SSE = 0.064, 0.02). Similar results can also be observed in the VMAT validation plan results (Figure 7c and Figure 7d). Additionally, for IMRT validation plans, the D0 and REF curves are highly overlapped (SSE = 0.074, 0.022) but there is a notable difference between them in VMAT validation plans (SSE = 1.976, 0.132).

The average and STD values of gamma passing rates of corrected displaced data, which are obtained by applying the correction model and specific correction matrices, are presented in Table 3. In the same table, the average and STD values of the gamma passing rate differences between the corrected displaced and original non‐displaced data are also shown. It is found that when the correction model is used, for VMAT validation plans, there are significant gamma passing rate differences between the data from the displaced EPID and the non‐displaced EPID. However, for IMRT validation plans, such differences are negligible. Besides, if specific correction matrices are applied, the gamma passing rates for the corrected data are relatively close to that of cases without EPID displacement, no matter whether VMAT or IMRT plans are used. The observation is further supported by the discrepancy between the displaced and corrected results, where the first situation shows larger discrepancies (0.041 ± 0.028, 0.049 ± 0.030), while the latter three exhibit minimal differences.

**TABLE 3:** Average and STD values of gamma passing rates of data corrected by correction models and specific correction matrices, as well as average and STD values of the gamma passing rate differences between the corrected displaced and original non‐displaced data.

Method	Correction model						Specific correction matrices			
Technique	VMAT			IMRT			VMAT		IMRT	
Displacement (cm)	0	2.2	3.7	0	2.2	3.7	0	3.7	0	3.7
Average	0.701	0.661	0.653	0.999	0.998	0.999	0.994	0.990	0.994	0.989
STD	0.064	0.055	0.053	0.002	0.002	0.002	0.010	0.011	0.007	0.011
D‐Average^a^	–	0.041	0.049	–	0.001	0.000	–	0.004	–	0.005
D‐STD^b^	–	0.028	0.030	–	0.001	0.001	–	0.009	–	0.004

*Note*: a: average values of the gamma passing rate differences between the corrected displaced and original non‐displaced data. b: STD of the gamma passing rate differences between the corrected displaced and original non‐displaced data.

**FIGURE 8:**
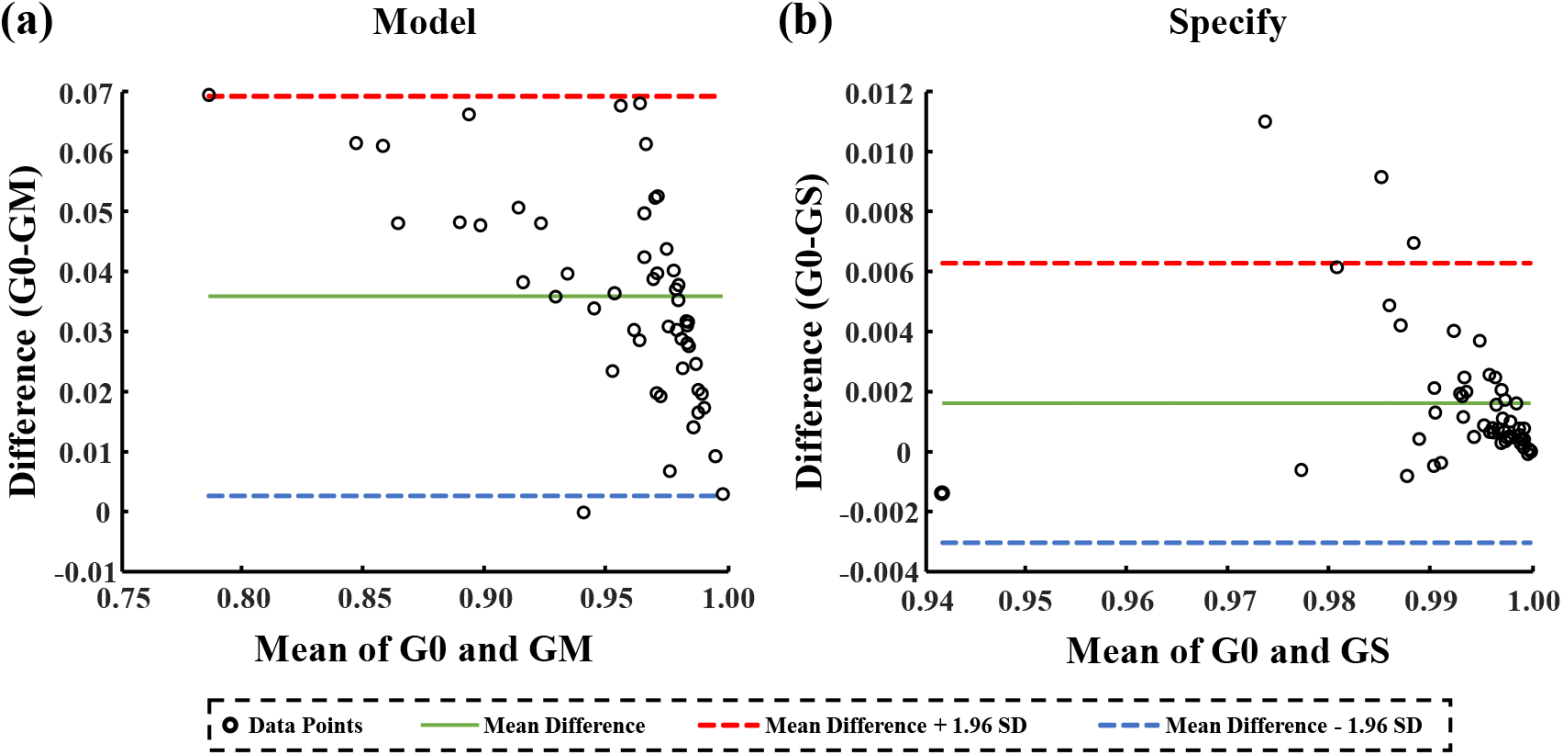
Consistency test results of the gamma passing rates between corrected data and non‐displaced data in Experiment No.5. (a) the correction model is used, (b) the specific correction matrices are used.

Figure 8 illustrates consistency test result of the gamma passing rates between the corrected displaced data and original non‐displaced data in Experiment No.5. The difference between gamma passing rates of non‐displaced data (G0) and data corrected by the correction model (GM) is more significant than the gamma passing rate difference between G0 and data corrected by specific correction matrices (GS) (the average difference of the former and the latter are 0.036 and 0.002). The scatter points basically fall within the 95% consistency interval (within 1.96 standard deviations [SD]), which demonstrate good consistency.

## 4 DISCUSSION

Building upon the findings from previous studies, it has been observed that the EPID displacements along the X‐axis and Y‐axis adhere to similar patterns. Consequently, the correction model for shifts along both the X and Y axes is interchangeable. In this paper, the correction model designed for displacements in the Y1 direction can be readily adapted for application in other three directions by employing certain transposition and data processing techniques. Alternatively, data collected from additional directions can be utilized to augment the correction model, enabling corrections in all four directions. In contrast, the directionality of specific correction matrices imposes no constraints.

As for the reason for the substantial errors observed in the data due to the displacement of the EPID panel relative to the calibration zero position, preliminary investigation reveals that the issue originates from the failure to assign values to the longitudinal and lateral parameters, which represents part of the location information of the EPID panel. When the PD verification plan is generated in the treatment planning system, only vertical parameters are assigned 0 in advance. Typically, when the plan is about to be executed, the treatment system assigns a value of 0 to longitudinal and lateral parameters, corresponding to the calibration center position of the EPID panel. However, for PD verification plans of certain patients with extra‐long target areas, where the treatment system determines that the irradiation range exceeds the maximum sensitive area of the EPID panel (43 cm × 43 cm), the precautionary measures will be carried out, to protect crucial electronics on the EPID. Such measures assign non‐zero values to longitudinal and lateral parameters, resulting in the displacement of EPID panels. This involuntary shift in hardware represents a necessary adjustment in the accelerator. Despite false alarms occurring occasionally and we can simply fix the EPID panel to its initial position based on clinical experience, it is undeniable that certain fields indeed pose a risk of irradiating and damaging the EPID electronics. In such cases, it becomes imperative to address the issue using the methods proposed in this study to finish PD verification through displaced EPID panels.

During the extension of the support arms of EPID, different backscatter effects of the EPID arms are introduced with the changes of displacements and according to the study on a‐Si 500 EPID,^4^ these effects also changes with the field sizes. To simplify the studied issues, the a‐Si 1200 EPID is used in this paper. Owning to the backscatter shielding material attached to the panel back,^3^ it shows significant dosimetric improvements and provides much more accurate measurements results than the previous a‐Si 1000, which has the same physical components as the a‐Si 500. Therefore, it is reasonable to believe that the backscatter effects of the arm has been reduced to to an acceptable level in this study.

Table 2 indicates that the correction matrix of the correction model exhibits a high degree of similarity to the actual correction matrix (gamma passing rate > 0.99), except the case where the field size is 12.5 cm × 12.5 cm (gamma passing rate = 0.968). After applying the correction model to actual verification plans, the gamma passing rates for non‐displaced VMAT verification plans are consistently higher than those for plans where the EPID is displaced by 2.2 cm and 3.7 cm. As shown in Table 3, the average values of such statistically significant differences are about 0.041 and 0.049 for different displacement distances, respectively. Similarly, IMRT verification plans with non‐displaced EPID exhibit higher gamma passing rates compared to those with EPID displaced by 2.2 and 3.7 cm, but significant differences are observed only between the plans with non‐displaced and 2.2 cm‐displaced EPID. Both for VMAT and IMRT verification plans, the gamma passing rates for plans, where EPID are offset distances of 2.2 and 3.7 cm, are remarkably similar, without significant differences between these two. This suggests that the correction model yields comparable results for different displacement distances of the EPID according to the field size. While there are differences in gamma passing rates between the corrected results and the non‐displaced results, the corrected outcomes are stable. Notably, the corrected results for IMRT verification plans closely resemble the non‐displaced results, which exhibit relatively larger disparities for VMAT verification plans instead. While C2.2, C3.7, and D0 share similar heights (Figure 5c and Figure 5d), the calculated gamma passing rates still exhibit significant disparities, which could potentially be attributed to the substantial inherent differences between D0 and REF. Given that D0 is obtained from collected data, it is plausible that the observed deviation in the gamma passing rates may stem from a bias in the PDIP algorithm's prediction of the REF dose for this VMAT plan.

Good consistency is observed in actual applications of the correction model between gamma passing rates of data corrected by the correction model and gamma passing rates of non‐displaced data (Figure 8a). Noteworthy, field sizes used here are smaller than the targets in IMRT plans due to the limitation of carriage, as shown in Figure 2b. This problem doesn't exist in IMRT plans for specific correction matrices and VMAT plans for both methods. However, concerning that the correction model proposed in this study is developed for square fields, its applications in IMRT plans are limited.

Another proposed method, the specific correction matrices, bypasses the correction model and collects corresponding open‐field data to generate matrices for data calibration. The results in Figure 6 show that, whether applied to VMAT or IMRT verification plans, the gamma passing rates for both non‐displaced and 3.7 cm‐displaced scenarios are highly similar when using specific correction matrices. Similar to the correction model, good consistency between gamma passing rates of the corrected and non‐placed data is also observed in the actual usage of specific correction matrices (Figure 8b). Therefore, results of Figure 6, Figure 7, Table 3, and Figure 8 indicate that specific correction matrices are effective and can serve as a method for data calibration when there is displacement in the EPID panel.

With the maturity of the jaw tracking feature, it has found widespread application in treatment planning. Compared with those applying static jaws, plans that utilize the jaw tracking feature can achieve better organ‐at‐risk protection with comparable target doses maintained, especially in reducing V5 effectively.^5^, ^6^, ^7^, ^8^, ^9^, ^10^ To simplify the process, the static jaw is adopted in the test of correction models and actual applications of specific correction matrices in this paper. Extending the usage of the correction model to jaw‐tracking plans is not feasible currently as it is only applicable to square fields. For correction matrices, this application means that specific matrices need to be developed for each subfield size and corresponding beam segments, which require extensive data collection efforts. Therefore, the current method is more applicable to plans utilizing static jaws. Although this requirement limits the usage of our methods on plans for organs‐at‐risk that are sensitive to low‐dose radiation, for those insensitive to low‐dose radiation, the calibration of verification plan data through our methods can still be achieved as the static‐jaw plans are devisable.

In recent years, the application of EPID analysis based on the inverse projection algorithm in the study of in‐vivo dosimetry during treatment has gained considerable attention.^11^, ^12^, ^13^ This approach allows for the verification of the patient's treatment position and the retrospective calculation of the dose delivered to the patient during the treatment process, showing promising prospects. However, when using the EPID panel at a greater source‐to‐surface distance during treatment, the issues explored in this study may arise, and the research findings presented could provide valuable insights.

## 5 CONCLUSION

The current methods involving correction model and specific correction matrices can correct the data collected from the displaced EPID, and the gamma passing rates of the corrected data show results that are comparable to some extent with those of non‐displaced data. Particularly, the results corrected by specific correction matrices closely resemble the data from non‐displaced EPID. However, it's important to note that the current methods are only applicable to plans with static jaws, and further research is needed to extend the application of these methods to jaw tracking plans. Moreover, the proposed correction model method can be improved to work for rectangular field sizes either, whose usages in IMRT plans are limited now as only square correction matrices are generated.

## AUTHOR CONTRIBUTIONS

Haibin Zeng conducted the data collection, analyzed the data and wrote the original draft. Jian Guo and Leyuan Zhou helped perform the analysis with discussions and edit the manuscript. All authors reviewed the manuscript. We confirm that all authors contributed to the study.

## CONFLICT OF INTEREST STATEMENT

The authors have no relevant conflicts of interest to disclose.

## Data Availability

The data that support this study are not open access but are available from the corresponding author upon reasonable request.

## References

[1] Ma Y , Wang X , Mai R , et al. An electronic portal image device (EPID)‐based multiplatform rapid daily LINAC QA tool. J Appl Clin Med Phys. 2021;22. doi:10.1002/acm2.13055 PMC7856503 33410254

[2] Vejdani NV , Shahrookh N , Kazem A , Maryam N , Mehdi M . Evaluation of set‐up errors and determination of set‐up margin in pelvic radiotherapy by electronic portal imaging device (EPID). J Radiotherapy Pract. 2019;19(2):150‐156. doi:10.1017/S1460396919000566

[3] Mhatre V , Pillakal S , Chadha P , Talapatra K . Dosimetric Comparison of a‐Si 1200 and a‐Si 1000 Electronic Portal Imager for Intensity Modulated Radiation Therapy (IMRT). J Nuclear Medicine & Radiation Therapy. 2018;09(1). doi:10.4172/2155-9619.1000354

[4] Rowshanfarzad P , McCurdy BM , Sabet M , Lee C , O'Connor DJ , Greer PB . Measurement and modeling of the effect of support arm backscatter on dosimetry with a varian EPID. Med Phys. 2010;37(5):2269‐2278. doi:10.1118/1.3369445 20527561

[5] Feng Z , Wu H , Zhang Y , Zhang Y , Cheng J , Su X . Dosimetric comparison between jaw tracking and static jaw techniques in intensity‐modulated radiotherapy. Radiation Oncology. 2015;10(1):28. doi:10.1186/s13014-015-0329-4 25623899 PMC4326511

[6] Mani KR , Muneem MA , Sultana N , et al. Dosimetric comparison of jaw tracking in intensity modulated and volumetric modulated arc radiotherapy for carcinoma of cervix. Polish J Med Phys Eng. 2019;25:155‐164.

[7] Mani KR , Upadhayay S , Das KJ . Influence of jaw tracking in intensity‐modulated and volumetric‐modulated arc radiotherapy for head and neck cancers: a dosimetric study. Radiat Oncol J. 2017;35(1):90‐100. doi:10.3857/roj.2016.02054 28395504 PMC5398351

[8] Thongsawad S , Khamfongkhruea C , Tannanonta C . Dosimetric effect of jaw tracking in volumetric‐modulated arc therapy. J Med Phys. 2018;43(1):52‐57. doi:10.4103/jmp.JMP_75_17 29628634 PMC5879824

[9] Wu H , Jiang F , Yue H , et al. A comparative study of identical VMAT plans with and without jaw tracking technique. J Appl Clin Med Phys. 2016;17(5):133‐141. doi:10.1120/jacmp.v17i5.6252 27685122 PMC5874095

[10] Yao S , Zhang Y , Chen T , et al. Dosimetric comparison between jaw tracking and no jaw tracking in intensity‐modulated radiation therapy. Technology in Cancer Research & Treatment. 2019;18:1533033819841061. doi:10.1177/1533033819841061 31014182 PMC6488724

[11] Olaciregui‐Ruiz I , Rozendaal R , RFMv Oers , Mijnheer B , Mans A . Virtual patient 3D dose reconstruction using in air EPID measurements and a back‐projection algorithm for IMRT and VMAT treatments. Physica Medica. 2017;37(04):49‐57. doi:10.1016/j.ejmp.2017.04.016 28535915

[12] Norimasa M , Mitsuhiro N , Makoto S , Shinsuke Y , Michio Y , Takashi M . Analyses of integrated EPID images for on‐treatment quality assurance to account for interfractional variations in volumetric modulated arc therapy. J Appl Clin Med Phys. 2020;21(1):110‐116. doi:10.1002/acm2.12805 31909889 PMC6964755

[13] Igor OR , Roel R , vK Simon , Ben M , Anton M . The effect of the choice of patient model on the performance of in vivo 3D EPID dosimetry to detect variations in patient position and anatomy. Med Phys. 2020;47(1):171‐180. doi:10.1002/mp.13893 31674038

